# Characteristic of phosphorus rich compounds in the incinerated sewage sludge ashes: a case for sustainable waste management

**DOI:** 10.1038/s41598-023-36407-7

**Published:** 2023-06-05

**Authors:** Monika Kasina, Kinga Jarosz, Mateusz Stolarczyk, Jörg Göttlicher, Ralph Steininger, Marek Michalik

**Affiliations:** 1 Institute of Geological Sciences, Jagiellonian University, Gronostajowa 3a, 30-387 Kraków, Poland; 2 Institute of Geography and Spatial Management, Jagiellonian University, Gronostajowa 7, 30-387 Kraków, Poland; 3 Institute of Photon Science and Synchrotron Radiation (IPS), Karlsruhe Institute of Technology (KIT), Hermann-Von-Helmholtz-Platz 1, 76344 Eggenstein-Leopoldshafen, Germany

**Keywords:** Environmental sciences, Materials science

## Abstract

Growing concern over mineral resources supply forces us to search for alternative sources of Phosphorus. The possibility to recover phosphorus from incinerated sewage sludge ashes appears to be an important aspect in anthropogenic phosphorus cycle and sustainable economy. To make phosphorus recovery efficient it is important to learn the chemical and mineral composition of ash and phosphorus speciation. The phosphorus content in the ash was over 7%, what corresponds to medium rich phosphorus ores. The main phosphorus rich mineral phases were phosphate minerals. The most widespread was tri-calcium phosphate Whitlockite with various Fe, Mg and Ca proportions. In minority Fe–PO_4_ and Mg–PO_4_ were detected. Whitlockite commonly overgrown with hematite, influences negatively mineral solubility and thus recovery potential and indicates low bioavailability of phosphorus. Considerable amount of phosphorus was found in the low crystalline matrix where phosphorus content was around 10 wt% however low crystallinity and dispersed phosphorus also does not strengthen the potential to recover this element.

## Introduction

Phosphorus is a fundamental macronutrient for life and growth on Earth^1^ including being a building substance of DNA and RNA^2^. Moreover, global food supply relies on it as a key resource for fertilizer production^3^. It is however a limited, non-renewable resource that cannot be replaced by any anthropogenically-synthetized substance^4^. It is estimated that it will run out completely, if the usage remains on the same levels as currently, within less than 300^2^ to 400 years worldwide^5^ and even faster on the regional scale, as mineral phosphorus deposits are not evenly distributed and their availability varies^6,7^. Therefore, as soon as 2014 phosphorous was recognized by European Commission as a critical raw material^8^. The phosphorus demand is constantly increasing with the population growth and development. At the same time due to low efficiency of phosphorus cycles causes significant losses (e.g., by improper fertilizer production and usage, inefficient mining and processing) and unwanted excesses in nature (often resulting in eutrophication)^9^. The issue of phosphorus sourcing and usage is therefore critical for the development, food supply resilience and pollution perspectives^10^.

A stable supply for phosphorus raw materials and its proper management is one of the major challenges for developing economies nowadays. There is an increasing attention on the development of strategies for phosphorus refining, recovery and reusing especially for industrial and agricultural purposes^11^.

The content of P_2_O_5_ in phosphate ores reaches from 2 to 6% for low grade ores and from 25 to 34% high grade ores, depending on the processing methods, mining, geological conditions and other factors. The incinerated sewage sludge ashes (ISSA) contain on average 14–25% P_2_O_5_^12^. These high concentrations allow to classify the ISSA as medium rich ores and consider it as a potential anthropogenic source of phosphorus. By ISSA usage it is possible to keep phosphorus in the production cycle by recycling it and simultaneously protect natural resources and hence fulfil the assumption of rational waste management and sustainable development. This aspect will be facilitated due to the fact that increasing amount of ISSA will be produced annually. This is related to the predicted growing amount of sewage sludge production as a result of world population growth and its incineration as one of the most effective method to reduce the amount of landfill waste. We also have to keep in mind that prices of ores can fluctuate and reach high levels especially for critical elements what will make easily available, price-competitive material such as ISSA very attractive.

The current major challenge in Phosphorus recovery from ISSA is associated with the economics of established processes such as acidic or alkaline leaching^13–15^. With a fine profitability margin the procedures applied need to be carefully designed and extremely efficient. To ensure quality and efficiency the material needs to be characterized in detail. There is a room for new methods, for example biobased^16^, using phosphorus-selective adsorbents coupled with acidic leaching^17^, or by introducing additives before the incineration process in order to change extraction efficiency by influencing mineralogical characteristic of incineration product^18,19^. Nevertheless, the first step toward effective element recovery still is to characterize the source material, focusing on the targeted element forms.

The sector greatly relying on Phosphorus supply is agriculture, therefore Phosphorus separation from heavy metals is of critical importance and ISSA cannot be safely applied without any further treatment, especially taking into consideration massive amounts of fertilizer being used in the long run. In experiments directly applying ISSA to soil the conclusion was that large amounts of ISSA need to be used due to low bioavailability and effectiveness is mostly depending on soil Phosphorus buffering characteristic and pH^20^. The further characteristic of Phosphorus bearing minerals in ISSA proves once again to be necessary to provide tailored solutions for specific conditions by for example introducing additives or plant microbiota for Phosphorus phases transformation, as well as applying ISSA, purified from heavy metals, as a fertilizer for specific crops.

Phosphorus recovery from ISSA has been a topic of growing scientific interest however, less research is focused on the characterization of phosphorus rich compounds which usually are present as complex aggregates, difficult to distinguish or present as amorphous phases whereas phosphate minerals are not common^4^. This makes the recovery of phosphorus difficult and often inefficient^21^, which is why the detailed characterization of phosphorus rich compounds is so important and of a big relevance in waste management and sustainability. The development of recovery methods, which are still not fully efficient even for the natural phosphorus resources, starts with good understanding of its occurrence and composition. Phosphorus, as a highly reactive element, does not occur in free elemental form. It could be found in different phosphorus-containing minerals, mostly phosphates^2^. Moreover, the form in which is present determines its potential as pollutant, and possibility for direct use as a substitute for synthetic fertilizer. There are many papers in the literature describing various methods of Phosphorus recovery and their efficiency (Ref.^16^ and reference therein) but P-rich phases are usually described in very general way. It is important to remember that mineral composition may influence in a significant way mobility and bioavailability of nutrients and therefore influence the effective usage of ISSA. For this reason a detailed mineral characterization of P-rich phases and P-bonds is extremely important and must not be omitted. In order to quantify and characterize phosphorus rich components various analytical methods were applied. Additionally, a phosphorus fractionation was determined in order to define bioavailability of the element present in ISSA.

## Materials and methods

### Sewage sludge incineration ash

In order to characterize the phosphorus containing components an incinerated sewage sludge ash (ISSA) was studied.

The ISSA was collected from the sewage sludge incineration plant located in the urban area in the south of Poland. All together six samples were collected in various periods (November 2015, March 2016, July 2016, December 2016, October 2017, March 2017) in order to control the variation in mineral and chemical composition and physical properties in time.

The ISSA is classified as non-hazardous waste (Guidance on classification of waste according to EWC-Stat categories, 2010 and Annex III to Directive 2008/98/EC)). It is a reddish in colour, fine-grained material with a dominance of 63 vol% of particles below 100 µm^22^.

### Analytical methods

#### Chemical and mineralogical methods

The content of elements in the ISSA was studied using inductively coupled plasma mass spectrometry (ICP-MS) and inductively coupled plasma atomic emission spectroscopy (ICP-AES). The loss on ignition (LOI) was performed to determine the volatile components and water content in the samples using thermal methods. Additionally sulphur and carbon content using LECO analyses were performed by Bureau Veritas Minerals in Vancouver, Canada.

A Hitachi S-4700 field emission scanning electron microscope (FE-SEM) coupled with a Noran energy dispersive spectrometer (EDS) was used for detailed microscopic observations and characterization of forms of occurrence of phosphorus rich components within the crystalline and amorphous components. Samples were prepared as both thin sections and grainy material attached directly to the carbon adhesive discs mounted on carbon holders, and carbon coated. A secondary electron (SE) and backscattered electron (BSE) imaging modes were used at accelerating voltage 20 kV and beam current 10 mA. Analyses were performed at the Institute of Geological Science at the Jagiellonian University (Kraków, Poland).

A Philips X’Pert diffractometer (APD type) with a goniometer PW 3020 (angle range of 2°–70° 2Θ with a step of 0.02° 2Θ s^− 1^) recorded using a CuKα X-ray source was used for qualitative and quantitative X-ray diffraction analyses (XRD). Identification of phase composition was preformed using Philips X’Pert software (based on the ICDD database). To quantify the content of mineral and amorphous components a Siroquant software packages (Sietronics Pty Ltd) were used. For the qualitative XRD analyses samples were averaged, and milled dry in an agate mill, whereas for the quantitative analyses 2.7 g of the sample with the addition of 0.3 g of ZnO (as the internal standard) was milled in McCrone micronizing mill (Glen Creston) with ethanol for 10 min. Measurements were performed at an angle range of 2°–70° 2Θ with a step of 0.02° 5 s^− 1^. Analyses were conducted at the Institute of Geological Science at the Jagiellonian University (Kraków, Poland).

In order to obtain detailed information on characterization of phosphorus rich components in ISSA micro-X-ray diffraction (µ-XRD) analyses were performed at the synchrotron radiation source of the KIT (Germany), former known as ANKA. The data were collected at the X-ray beamline of the Synchrotron Laboratory for Environmental Studies (Synchrotron Umwelt-Labor SUL-X). Analyses were performed on the grains attached to the foils previously detached from the thin section supporting glass. A Si(111) crystal pair with a fixed beam exit was used as monochromator. The µ-XRD data were acquired with a CCD detector (Photonic Science XDI VHR-2 150) in symmetric transmission mode at 17 keV (0.72887 Å) with a beamsize at sample position of about 50 µm (hor.) × 50 µm (vert.). Detector—sample distance and energy were calibrated using NIST 660b LaB6. CCD frames with the Debey rings were radially integrated with the FIT2D software^23^ and as 1D X-ay diffractograms further processed for mineral phase identification by the Bruker Diffrac Plus—Eva program including the ICDD PDF database^24^.

#### Sequential phosphorus fractionation method

A fractionation of the phosphorus was conducted using modified Hedley method^25^. For this analysis 0.5 g of ash sample was used, in which the phosphorus forms were sequentially extracted using a series of reagents (sample/solution ratio 1:100). Readily soluble phosphorus forms (i.e., labile P) were extracted with distilled water (soluble phosphorus—PH_2_O) as well as 0.5 M NaHCO_3_ (i.e., exchangeable mineral phosphorus—PHCO_3_), followed by moderately available phosphorus forms (i.e., moderately labile P) which were extracted using 0.1 M NaOH (i.e., mineral P bound to Al and Fe oxides—PNaOH) and 1 M HCl (i.e., mineral P bound to calcium—PHCl). The most stable phosphorus form (i.e., stable P) was extracted using 12 M HCl with heating (i.e., poorly soluble mineral PHClconc). The content of each phosphorus fraction was determined spectrophotometrically using SPECORD 50 (Analytik Jena) via the chlorostannous acid method^26^. The experiment was performed at the Institute of Geography and Spatial Management at the Jagiellonian University (Kraków, Poland).

#### Enrichment methods

Additionally, basic sorting methods used in ores and mineral industry were performed in order to enriched material and get the overview on the possibility to separate valuable components from the ISSA. These included magnetic separation using neodymium magnets, concentration table and flotation. Analyses were performed at the University of Science and Technology AGH (Kraków, Poland).

## Results and discussion

### Characterization of ISSA

The content of phosphorus in the studied samples varied between 7.02 and 7.81 wt% (16.10 and 17.92 wt% of P_2_O_5_). It is within the range of medium rich ores^22^. From this point of view P recovery from ISSA seems to be feasible and practical therefore, we may consider ISAA as a waste-based source of this element. The averaged content of other major and trace elements present in the ash is listed in Table 1. It is Si–Fe–P–Ca dominated material with lower content of Al and Mg (few wt%) and LOI (2.7 wt% av.). These major compounds in ISSA are non-hazardous^27^ and ensure sustainable usage of ashes in waste management, where they can be used as a secondary material used in the cement production, concrete additives, ceramic and tiles production, building and construction industry^27–29^, and thus fulfilling the assumption of closed-loop economy.

**Table 1:** The averaged concentration of major and minor elements present in the ISSA.

Element		Averaged concentration
SiO_2_	%	37.65
Si		17.58
Al_2_O_3_	%	8.26
Al		4.37
Fe_2_O_3_	%	14.17
Fe		9.91
MgO	%	3.56
Mg		2.15
CaO	%	11.70
Ca		8.36
Na_2_O	%	0.69
Na		0.51
K_2_O	%	1.86
K		1.54
TiO_2_	%	0.94
Ti		0.56
P_2_O_5_	%	17.20
P		7.50
LOI	%	3.283
As	mg kg^−1^	14.23
Cd	mg kg^−1^	5.97
Cr	mg kg^−1^	711.36
Cu	mg kg^−1^	632.58
Mn	mg kg^−1^	0.08
Mo	mg kg^−1^	21.72
Ni	mg kg^−1^	103.97
Pb	mg kg^−1^	138.78
Zn	mg kg^−1^	3975.83
∑REE	mg kg^−1^	100.29
C_tot_	%	0.14
S_tot_	%	0.69

The total C and S contents (wt%) were low; 0.14 and 0.7 respectively.

There are many factors that can affect phosphorus precipitation such as basic pH and the presence of calcium carbonates^30^. A high calcium content may negatively influence the phosphorus availability^31^ as well as basic pH and thus influence the phosphorus release from the ISSA. Precipitation or presence of calcium carbonate in the system causing reduction of free calcium concentration required for the phosphate precipitation. Nevertheless, the Ca/P ratio was lower than one in the studied samples indicated that this problem will not occur. In addition Anawati and Azimi^32^ pointed out that high Al+Fe may influence negatively the phosphorus recovery by precipitation of insoluble salts. The low S content exclude the sulphide precipitation over (hydr)oxides precipitation which is characterized by better solubility in the environment^33,34^. From the environmental point of view precipitation of sulphide minerals would be advantageous, since it is responsible for bonding and immobilization of metals considered as toxic^35^ such as As, Zn, Cu, Cd and which presence may also negatively influence the phosphorus recovery^32^. From our previous study^21,36^ we learned that the chelating Na-EDTA compound indicated low ability to extract phosphorus (at the level of only 35%), and the highest ability to extract heavy metals and potentially toxic elements (As, Zn, Mo) whereas the greatest release of phosphorus was obtained using sulphuric acid at the level of 64% however obtained fraction is considered non-bioavailable^37^.

**Figure 1:**
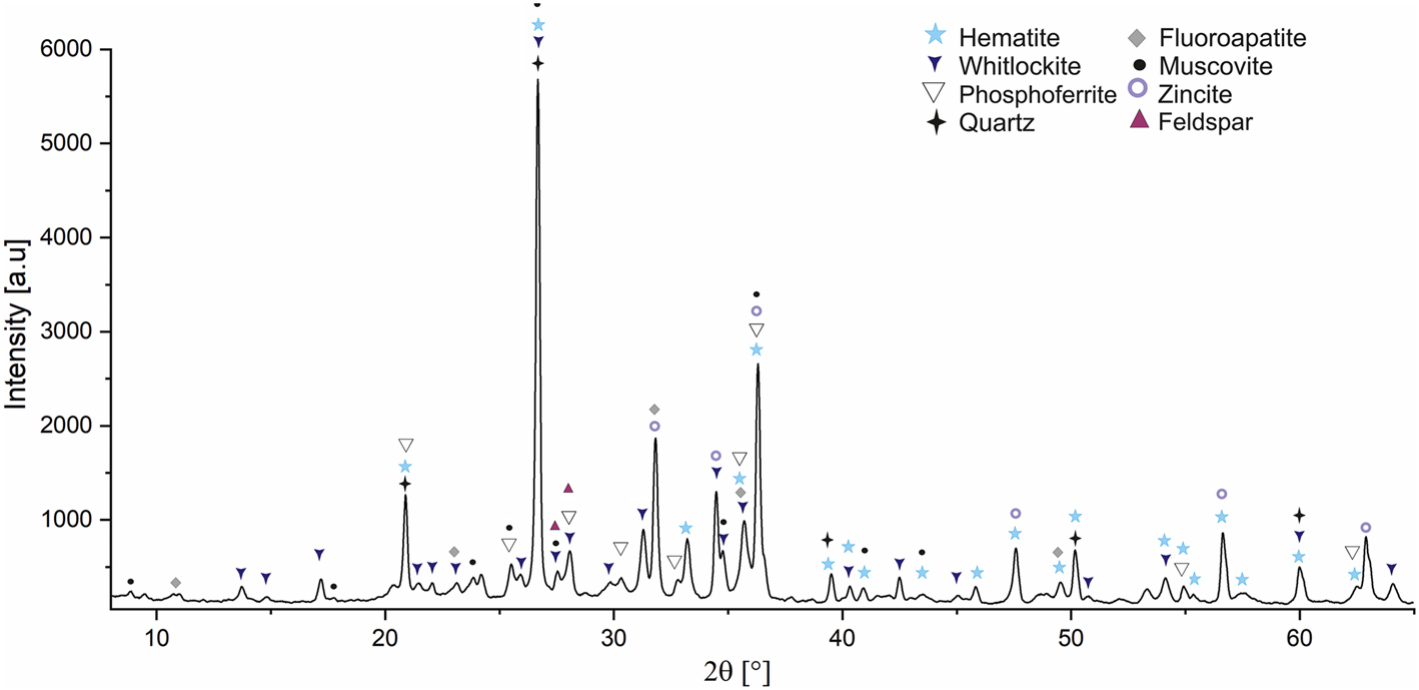
The XRD pattern of ISSA sample.

Figure 1 shows the exemplary XRD pattern of studied ISSA. The main crystalline phases were identified as quartz, feldspar, muscovite, hematite, whitlockite and Fe–PO_4_. Mineral phases such as quartz, feldspar and muscovite were of detrital origin. More likely they represent the components present in the street dust.

A slightly elevated background indicated either poorly crystalized phases or presence of amorphous phase. The quantitative analyses using Rietveld refinement estimated the content of amorphous phase at 19.9 ± 2.4 wt%. It was not possible to identify other crystalline phases by matching small peaks.

The whitlockite (Ca_9_(Mg,Fe)(PO_4_)6PO_3_OH), was the dominant phosphorus mineral phase in studied ISSA (22 ± 0.8%). Due to large similarity in diffraction peak position, it was not possible to distinguish using XRD if the whitlockite was represent the pure tri-calcium phosphate or with partial Fe, or Mg substitution for Ca. Adam et al.^12^ made a suggestion that Ca^2+^ in Whitlockite might be partially substituted with Mg^2+^, Fe^3+^ or Al^3+^. The EDS analyses of our samples indicated various proportion in Ca, Mg and Fe content. The lower content of Ca in the EDS analyses was 0.64 wt% and the highest was above 1.45 wt%. Mg was 1.94 and 5.68 respectively, whereas in case of Fe the variations were the largest form Fe free whitlockite to Fe reaching up to 61.45 wt%.

A very specific conditions during the incineration of sewage sludge (incineration in the fluidised bed boiler (Pyrofluid™), which operates at a stable operating temperature in the range of 850–900 °C^22^) and presence of Ca, Mg, Fe and P in the system seem to favor whitlockite formation over other phosphates (which are present only in minority). According to Donatello and Cheeseman^28^ and Choi et al.^38^ phosphate concentrates in the ISSA in the form of Whitlockite that is thermally stable and does not volatilize both during sludge drying and incineration process.

**Figure 2:**
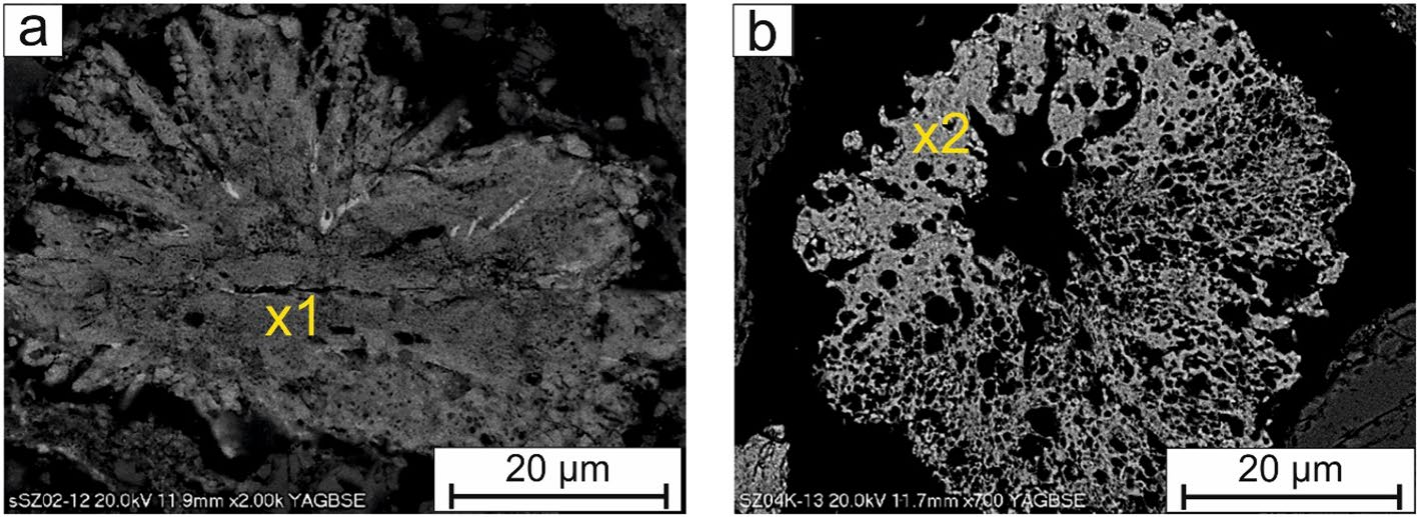
(**a,b**) Radially shaped whitlockite. SEM-BSE images (x1, x2—analytical points).

**Figure 3:**
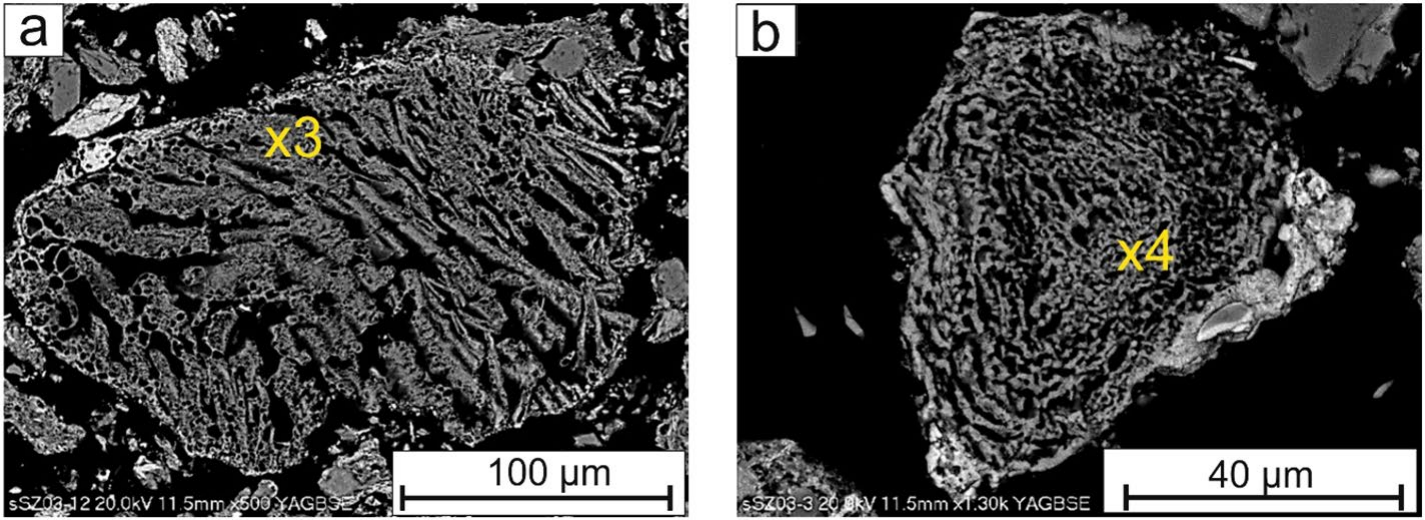
(**a**) Whitlockite crystals in the form of prisms and (**b**) in the form of rosette. SEM-BSE images (x3, x4—analytical points).

**Figure 4:**
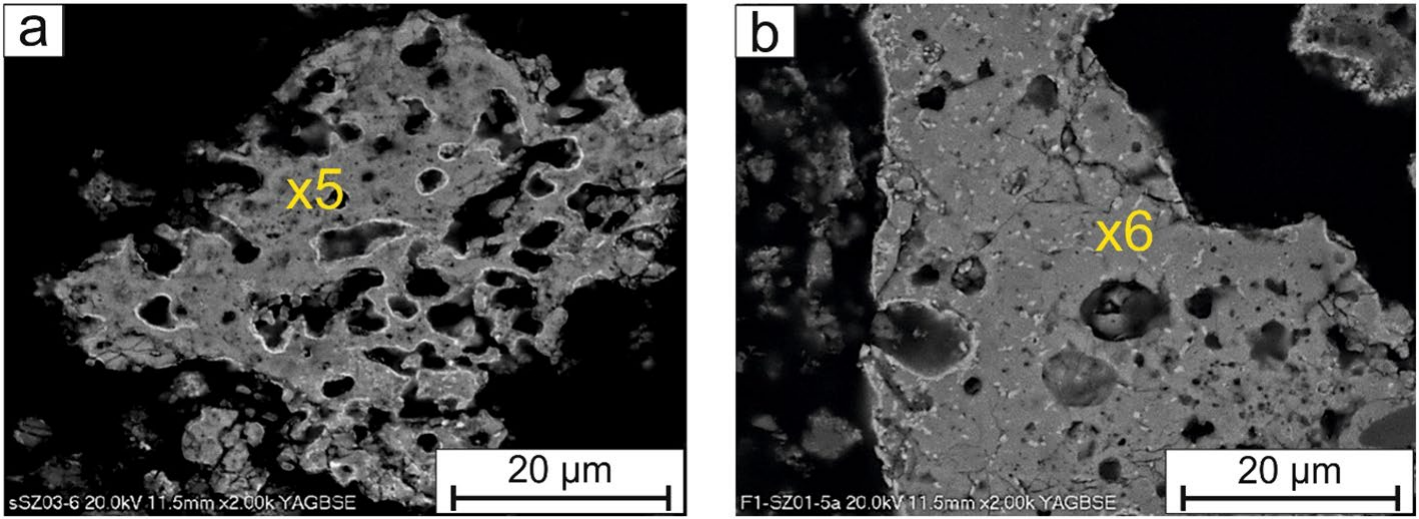
(**a,b**) A Moon-like sinters composed of whitlockite. SEM-BSE images (x5, x6—analytical points).

**Figure 5:**
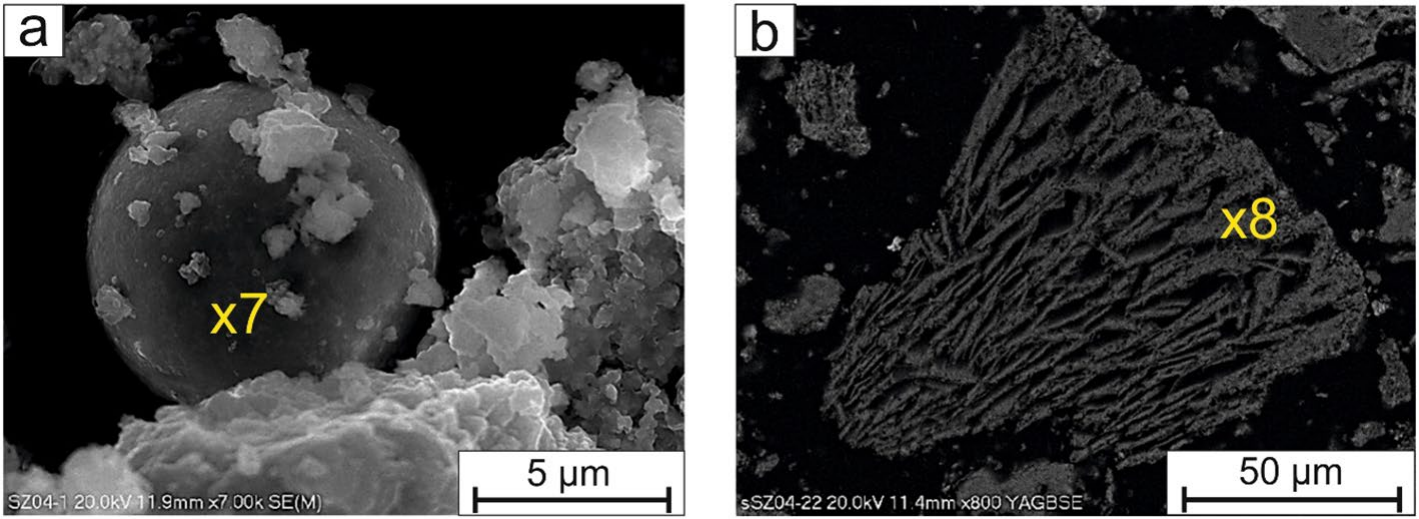
(**a**) Rounded crystal of Fe–PO_4_. SEM-SE image; (**b**) Magnesium rich phosphate in the form of prisms. SEM-BSE images (x7, x8—analytical points).

The phosphates of whitlockite group were radially shaped (Fig. 2a,b), rosette-like (Fig. 3a,b) or sintered with a “moon-like structure” (Fig. 4a,b), porous crystals with a size of up to 50 µm. The Fe–PO_4_ were usually perfectly rounded (Fig. 5a), however irregular sintered crystals were also found.

Additionally single Mg–phosphates were detected, usually in the form of prisms (Fig. 5b) or rosette-like (Fig. 7b). Mg-rich phosphates were larger than 100 µm.

**Figure 6:**
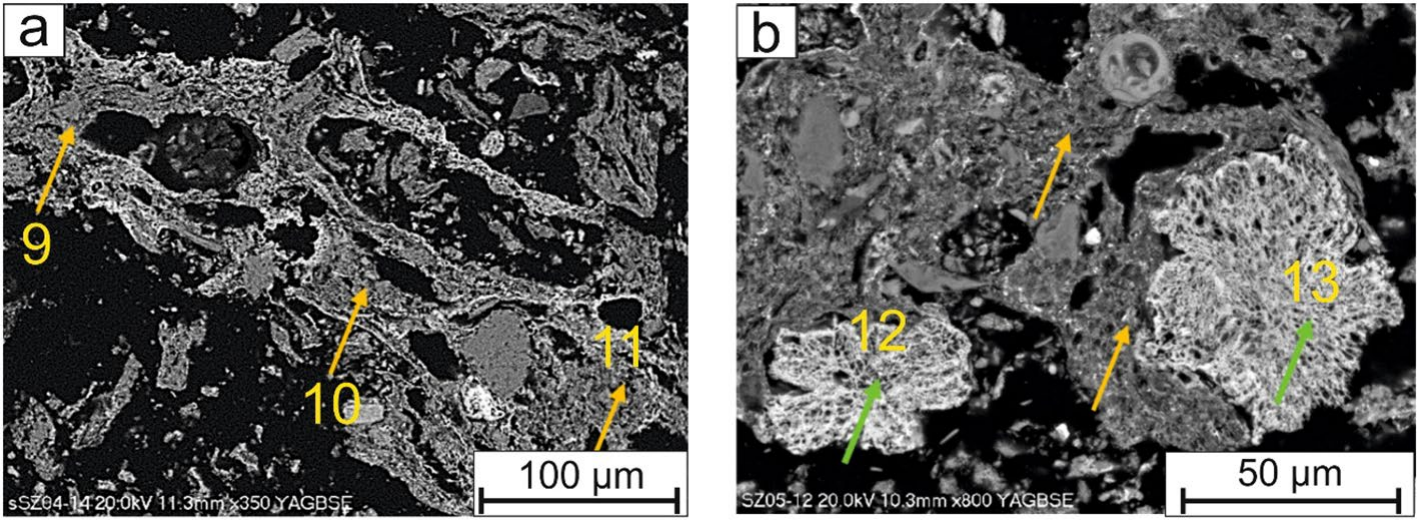
(**a,b**) Phosphate rich matrix (yellow arrow), surrounding rosette whitlockite rich in Fe (green arrow). SEM-BSE images (9, 10, 11, 12 and 13—analytical points).

Phosphorus was also detected in a matrix (Fig. 6a,b), a shapeless assemblages composed of Si, Al, Fe, Ca, P, Mg, K, Na where P content was on average 10 wt%.

**Figure 7:**
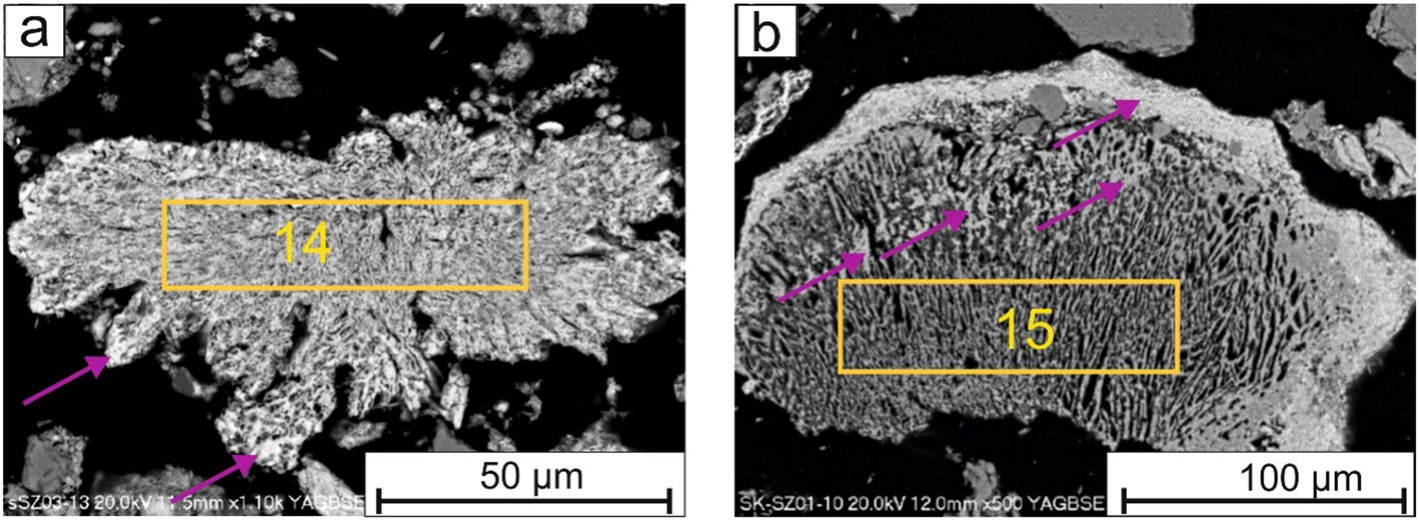
(**a**) Overgrowth of hematite (pink arrow) in whitlockite (yellow rectangle) and (**b**) in magnesium rich phosphate (yellow rectangle). SEM-BSE images (14, 15—analytical points).

Commonly, pores in whitlockite were filled or overgrown with hematite crystals (Fig. 7a). Also, at the edges of phosphorus rich minerals hematite coatings are present (Fig. 7b).

**Figure 8:**
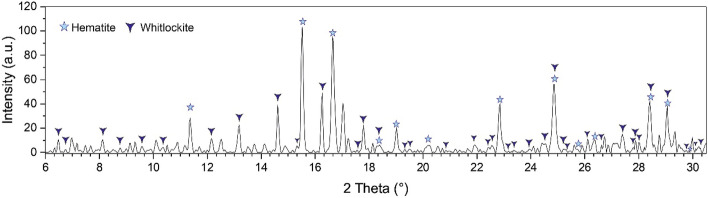
An exemplary, slightly smoothed µXRD pattern of studied phosphorus-rich mineral (λ = 0.72887 Å), showing hematite (according to the ICDD PDF 00-033-0664) and whitlockite (according to the ICDD PDF 01-087-1582) as main mineral phases.

The composition of selected phosphorus rich components based on EDS analyses is shown in the Table 2. The analytical points are marked in the Figs. 2, 3, 4, 5, 6 and 7. In addition, the results of µXRD indicated the presence of microcrystals of hematite in whitlockite (Fig. 8) as separate phase, and not as previously thought as Fe substituting calcium in whitlockite structure. It seems that both phases were formed during the ash incineration inside the fluidized bed reactor and neither as the result of other minerals transformation under the high temperatures nor as phases present in the sand used for fluidization of the or contaminants from neighboring areas. It is a very important finding, since Fe is known to limit phosphorus recovery (e.g.^39^), when significant amount of phosphorus is bond to Fe. Interestingly, there was no correlation between morphology of P-rich phases and their composition.

**Table 2:** The chemical composition of phosphorus rich phases present in the Figs. 2, 3, 4, 5, 6 and 7 based on EDS analyses.

Analytical point	1	2	3	4	5	6	7	8	9	10	11	12	13	14	14
Wt%															
O	28.54	19.58	39.90	42.97	42.21	21.02	32.94	25.64	24.96	26.62	26.38	12.86	16.68	42.28	21.95
Na	1.08				0.30				0.45			0.56			0.56
Mg	**5.81**	**2.81**	**7.29**	**3.55**	**3.27**	**2.26**	**2.33**	**21.94**	**5.71**	**3.00**	**2.59**	**3.52**	**5.43**	**4.74**	**24.07**
Al							8.60		8.81	5.17	7.15				
Si	0.49	0.88	0.35	7.35	0.26		12.29	1.73	20.35	8.51	10.89		0.43	0.15	0.33
P	**24.71**	**25.20**	**15.03**	**12.59**	**20.83**	**24.22**	**13.14**	**46.67**	**6.92**	**20.28**	**18.97**	**27.45**	**29.22**	**19.09**	**42.29**
S				0.69			1.56		0.48	0.48					
Cl			0.34								1.19	0.29	0.48	0.33	1.89
K			0.20	1.11			2.81		0.74	1.65	2.10	0.39	0.42		
Ca	**2.71**	**0.78**	**1.33**	**15.02**	**1.92**	**1.62**	**9.46**	**0.52**	**29.34**	**19.27**	**14.17**	**0.80**	**1.03**	**0.79**	**0.70**
Ti				0.59						0.86	2.04				
Mn	0.41		0.32	0.31								0.53	0.55		
Fe	**36.25**	**50.76**	**34.88**	**10.50**	**30.20**	**50.89**	**13.67**	**4.49**	**2.24**	**14.16**	**14.52**	**53.61**	**45.75**	**32.62**	**8.22**
Cu							1.57								
Zn				1.50			1.62								

Significant values are in bold.

Additionally, Fe is characterized by low solubility especially in basic pH^40^. Kasina^21^ indicated using sequential extraction method based on Golterman procedure^41^ the total recovery of phosphorus of around 40–60%, which compared to other described in the literature ISSA was very low. The recovery rates from ISSA can reach up to 90%^42^ depending on the ash composition. It is therefore important to treat ISSA form different localities in an individual way, since in different locations they may differ in composition and properties, thus strongly influence their recovery potential and usage.

The moon-like structure could be compared to the bubble microstructure described in Cheeseman et al.^43^, which more likely was obtained as a result of firing a sewage sludge in the temperatures higher than those required to obtained maximum density. That caused softening of glassy phase and removal of gasses gathered as a result of decomposition of inorganic phases present in sludge and pores formation.

It is highly probable that sintered and “closed” with hematite phosphate is also not easily susceptible to leaching and thus with lower recovery potential.

### Phosphorus fractionation and bioavailability

The result of sequential extraction allowed to determine the phosphorus content in five individual fractions (Table 3).

**Table 3:** Phosphorus fractionation and bioavailability.

Sample		Phosphorus fraction				
		Labile P		Moderately labile P		Stable P
		PH_2_0	PHCO_3_	PNaOH	PHCl	PHCl conc
SZ01	P [mg/g]	0.71	2.92	8.27	4.93	2.49
	% P	3.67	15.12	42.82	25.53	12.86
SZ03	P [mg/g]	0.45	3.33	7.65	4.31	3.80
	% P	2.30	17.04	39.16	22.07	19.43
SZ04	P [mg/g]	0.73	3.22	8.92	4.01	3.91
	% P	3.51	15.47	42.92	19.29	18.81
SZ022	P [mg/g]	0.64	3.75	9.02	2.52	4.29
	% P	3.19	18.53	44.60	12.48	21.20

SZ01–SZ022—samples name.

The highest concentrations were obtained for phosphorus bond to iron and aluminium oxides and calcium, which is in agreement to mineralogical observations and composition of the most common phosphate mineral in studied samples—whitlockite. Here, also the solubility of phosphorus bonds is of a great importance. Kim and Lee^44^ estimated using Visual MINTEQ software that phosphorus bond with calcium is more soluble than phosphorus bond with aluminium and iron at neutral pH, whereas phosphorus bond with calcium is very soluble in acidic conditions and practically insoluble in basic environment, on the contrary to phosphorus bearing aluminium and iron which are very soluble in both acidic and basic conditions and insoluble at neutral.

Over 15% of phosphorus were classified as exchangeable mineral phosphorus. Also, high concentrations were measured for phosphorus present in so called stable form. Only few % of phosphorus was released in the reaction with deionised water.

Additionally, apart from extracting particular fractions of phosphorus, we could determine the bioavailability of this element in the environment: labile—which is easily available to plants, moderately labile—where certain conditions such as high or low pH must occur to make the phosphorus available, and stable phosphorus which is completely immobilized and is not up-taken by plants. Moderately mobile phosphorus predominates in all samples. Here we may conclude that whitlockite components and whitlockite–hematite overgrowth are responsible for low solubility and rather low bioavailability of these minerals. We could also observe slight changes in proportion of liable versus stable phosphorus in time and decrease in concentration of phosphorus linked to moderately liable fractions.

### Enrichment of ISSA in phosphorus using ore processing technologies

In order to achieve enriched in phosphorus samples a possibly suitable ore processing technologies such as magnetic roll separator and flotation and concentration table methods were applied (Table 4). Magnetic separation was used to separate unwanted components such as heavy metals and to enriched phosphorus concentration in non-magnetic fraction. Interestingly, the ISSA did not separate in the magnetic separators, even though the pre-treatment with neodymium magnet caused attracting the material in the sample bag. Franz^4^ in his studies also found difficulties in obtaining phosphorus enriched material in magnetic separators.

**Table 4:** Material yields using different ore processing methods.

Grain size	Tool	Product	Share [%]
Natural	Concentration table	Concentrate	5.9
		Waste	94.1
Natural	Flotation 1	Concentrate	5.4
		Waste	94.6
	Flotation 2	Concentrate	3.0
		Waste	97.0

Moreover, flotation, which is widely used in the ore industry for processing apatite ores, failed to enrich phosphate constituents from SSA in the flotation froth^4,45^. In our experiments not only flotation but also separation using concentration table failed (Table 3). The yields for both sample treatment methods were on the level of a percent. In addition, similar concentrations were measured both in the concentrate and in the residual material. In addition, flotation caused a relative concentration of finer-grained material whereas concentration table caused the removal of fine-grained material.

**Figure 9:**
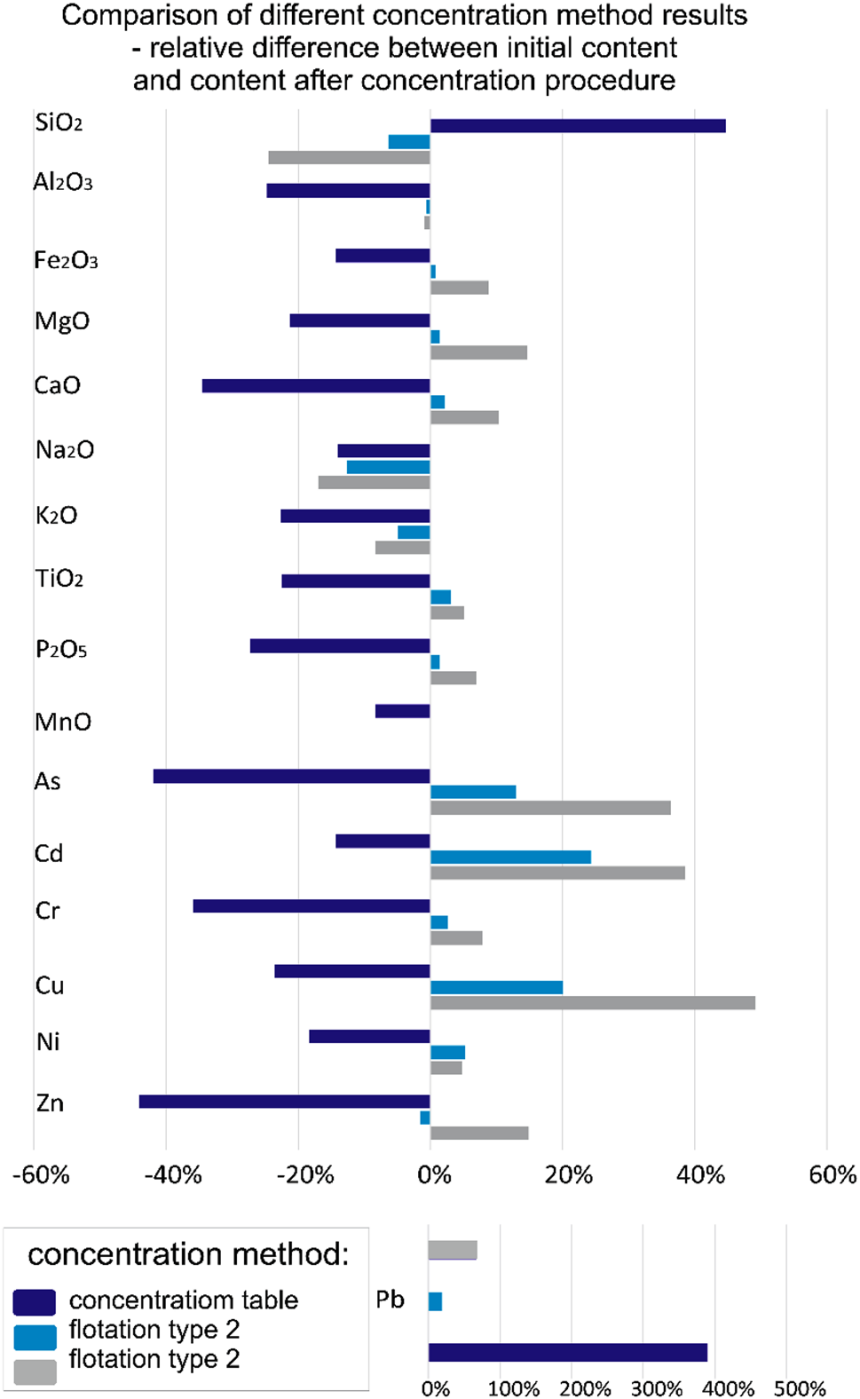
The enrichments efficiency of the exemplary ISSA sample.

The yields obtained for other main elements were also unsatisfactory, and did not exceed few wt% (Fig. 9).

The enrichment efficiency of minor elements, including heavy metals and potentially toxic elements for environment was moderate. Lead was heavily concentrated as a result of concertation table usage. As a result of flotation molybdenum, zinc, chromium and copper were slightly concentrated. Interestingly flotation 2 resulted in enrichment of particularly all elements (Fig. 9). Both, the concentration table and the flotation separation methods were ineffective for rare earth elements (Fig. 9).

## Conclusion

- The main crystalline phosphorus rich mineral phase was whitlockite, tri-calcium phosphate with various Fe, Mg and Ca proportions, and in minority also Mg, and Fe phosphates were present.
- Common were the overgrowths of whitlockite and hematite, and not as previously thought iron-rich whitlockite. This finding is important in terms of phosphorus recovery. Iron is known to influence negatively the phosphorus recovery potential.
- Presence of whitlockite and whitlockite-hematite overgrowths are responsible for low solubility and phosphorus release and thus also negatively influence bioavailability.
- Phosphorus was also dispersed within amorphous multi-elements matrix.
- Moderately mobile phosphorus predominates in all samples. Here we may conclude that whitlockite components and whitlockite–hematite overgrowths are responsible for low solubility and rather low bioavailability of phosphorus.

## Data Availability

All data generated or analysed during this study are included in this published article. The raw datasets used and/or analysed during the current study available from the corresponding author on reasonable request.
